# The role of general practice in the identification of age-related vision impairment and chronic eye diseases: a systematic review

**DOI:** 10.3399/BJGPO.2025.0021

**Published:** 2025-12-19

**Authors:** Marie Honoré Jacobsen, Olivia Hjulsager Mathiesen, Emma Katrine Frøhlke Steinbo, Agnes Galbo Brost, Frans Boch Waldorff, Catharina Thiel Sandholdt

**Affiliations:** 1 Research Unit for General Practice and Section for General Practice, Department of Public Health, University of Copenhagen, Copenhagen, Denmark; 2 National Research Centre for the Working Environment, Copenhagen, Denmark; 3 Danish Dementia Research Centre, Copenhagen, Denmark; 4 Department of Ophthalmology, Copenhagen University Hospital Rigshospitalet-Glostrup, Copenhagen, Denmark

**Keywords:** eye problems, systematic review, care of older people, general practice, primary health care

## Abstract

**Background:**

Age-related vision impairment (ARVI) is associated with an increased risk of dementia and depression and can affect older patients' overall health and ability to manage everyday tasks. ARVI is often asymptomatic making it difficult to detect. The World Health Organization (WHO) recommends primary care settings for identification of ARVI, underscoring the importance of general practice.

**Aim:**

To synthesise recent knowledge on identifying ARVI in general practice within countries with well-established primary healthcare systems.

**Design & setting:**

A systematic literature review searching for published research focused on identification of ARVI and chronic eye diseases in general practice.

**Method:**

The search was conducted in June 2024 across PubMed, Web of Science, and Scopus. Inclusion criteria included empirical, peer-reviewed studies focused on ARVI or eye diseases in adults in general practice, conducted in countries with well-established primary healthcare systems, and published in English or three Scandinavian languages (Danish, Swedish, and Norwegian). Acute eye diseases were excluded. Twenty articles were included. A thematic qualitative synthesis of included articles was conducted.

**Results:**

The following three themes were identified: (1) general practice screenings and referrals, highlighting a limited knowledge of eye health, but a high focus on diabetic retinopathy (DR); (2) collaboration between general practices and other health professions, implied the importance of cross-sectorial collaboration; and (3) potentials in general practice for detecting ARVI, through initiatives such as continued professional development, systematic DR screening, and more focus on other eye diseases than DR.

**Conclusion:**

This review highlights the need for more research in detection of ARVI and prevalent chronic eye diseases in general practice.

## How this fits in

Age-related vision impairment (ARVI) is associated with an increased risk of dementia and depression and can affect older patients' overall health and ability to manage everyday tasks. ARVI and chronic eye diseases can often be treated, or the progression of vision loss can be suspended if detected. ARVI is thus a relevant health issue for general practice to address with older patients. This systematic literature review synthesises the latest knowledge on the GP’s role in detecting ARVI and chronic eye diseases. The findings show limited research in the field and that the existing literature primarily focuses on diabetic retinopathy (DR). More research is needed on how ARVI can be expediently addressed in general practice.

## Introduction

ARVI is a public health concern posing a threat to the individual, family, and community as it can lead to loneliness, cognitive impairment, depression in both the patient and their relatives, diminished ability to self-care, reduced compliance to treatment plans, increased risk of falls and fractures, as well as lost earning capacity.^1–12^ARVI caused by presbyopia and moderate-to-severe ametropia (for example, myopia, hypermetropia and/or astigmatism) are frequent with 1.8 billion and 123.7 million people affected, respectively.^13^ These conditions can be corrected with optometry. The most common diseases that cause ARVI are age-related macular degeneration (AMD), glaucoma, cataract, and DR. In 2019, the World Health Organization (WHO) estimated that 146 million people live with ARVI caused by DR, 76 million by glaucoma, 65.2 million by cataract, and 10.4 million by AMD.^13^ WHO recommends vision screening every 1–2 years in individuals aged >50 years in a primary care setting.^14,15^ In health systems with a well-managed primary care sector, the GP plays an important role in identifying, treating, and managing the patients’ overall conditions and is typically the first health professional patients consult.^16,17^ Early detection and initiation of treatment for AMD, glaucoma, and DR is crucial to prevent disease progression and non-treatable vision loss.^13,18^ Unlike acute eye-conditions — such as detached retina, which will often present with clear symptoms for general practice to react on — symptoms for chronic, age-related eye diseases can be difficult to detect as they are often asymptomatic and patients may perceive changes in vision as part of a natural ageing process and not seek necessary help.^19^ Given the consequences of ARVI in older patients, addressing ARVI is a potential task for general practice owing to their responsibility of longitudinal continuity of care for the overall health status of the patient. The aim of this review is therefore to explore recent knowledge on identifying ARVI in general practice within countries with well-established primary healthcare systems.

## Method

### Search strategy

We follow the Preferred Reporting Items for Systematic Review and Meta-Analysis (PRISMA) 2020 guidelines.^20^ A pilot search conducted in PubMed in June 2022 indicated relevant literature. The final search on PubMed, Scopus, and Web of science was performed on 26 June 2024 using advanced search functions and limited to search in title, abstract, and keywords, see Table 1: search string. A filter for the past 11 years was used to optimise relevance. We included major age-related and chronic eye diseases: AMD, glaucoma, and cataract in the search string, as well as diabetic eye conditions, since diabetes is typically managed in general practice. These conditions are particularly relevant, as they can be missed during the treatable period owing to their slow progression. We excluded acute eye conditions with clear symptoms, such as iridocyclitis, retinal detachment, and amaurosis fugax, in the search string. This is owing to our aim of identifying existing research on ARVI including chronic eye diseases.

**Table 1.** Search string

Geographic area		Setting or population		Vision impairment		Detection
Sweden OR Norway OR Denmark OR nordic OR Europe* OR Canada OR Australia OR “New Zealand” OR England OR “United Kingdom” OR Ireland OR Germany OR Holland OR Netherlands OR Belgium OR Swedish OR Norwegian* OR Danish OR British OR Irish OR Finnish OR German* OR Canadian* OR Dutch OR Belgian*	**AND**	“General Practitioners” OR “General Practice” OR “General Practi*” OR “general practition*” OR “general practice*” OR “Family practic*” OR “Family physician*” OR “Family medicine”	**AND**	“eye disease*” OR “low vision” OR “Macular degeneration” OR “AMD” OR “Cataract” OR “Glaucoma” OR “Diabetic retinopathy” OR “Myopic degeneration” OR”(Diabetic eye disease” OR “Eye disease” OR “Vision loss” OR “Visual loss” OR “visual impair*” OR “visually impair*” OR “Vision impair*” OR “Low-vision” OR “Visual disability” OR “Visual disorder*” OR “Ophta*” OR “Ophtha*”	**AND**	identif* OR detect* OR diagnose* OR manage* OR “patient support*” OR role* OR support* OR screening* OR “case finding”

The search in PubMed included the following MeSH-terms: *Geographic area*: Europe, Canada, Australia, New Zealand. *Setting or population*: general practitioners and general practice. *Outcome:* vision disorders and eye diseases.

For the data selection process and criteria, see Table 2.

**Table 2.** Data selection process and criteria

**Screening method**	Covidence software			
**Inclusion criteria**	Empirical, published in a peer-reviewed journal, and had a human population	Reported on identification of vision impairment and/or chronic eye diseases in adults	Included general practice in countries with well-established primary healthcare systems	Available in English or Scandinavian languages
**Exclusion criteria**	Reported on acute eye issues	Did not include GPs or general practice personnel		
**Data selection process**	Identified articles: 441	Duplicates Removed: 162	Title and abstract screened: 279 and full-text screened: 40	Finally included articles: 20

**Figure 1.**
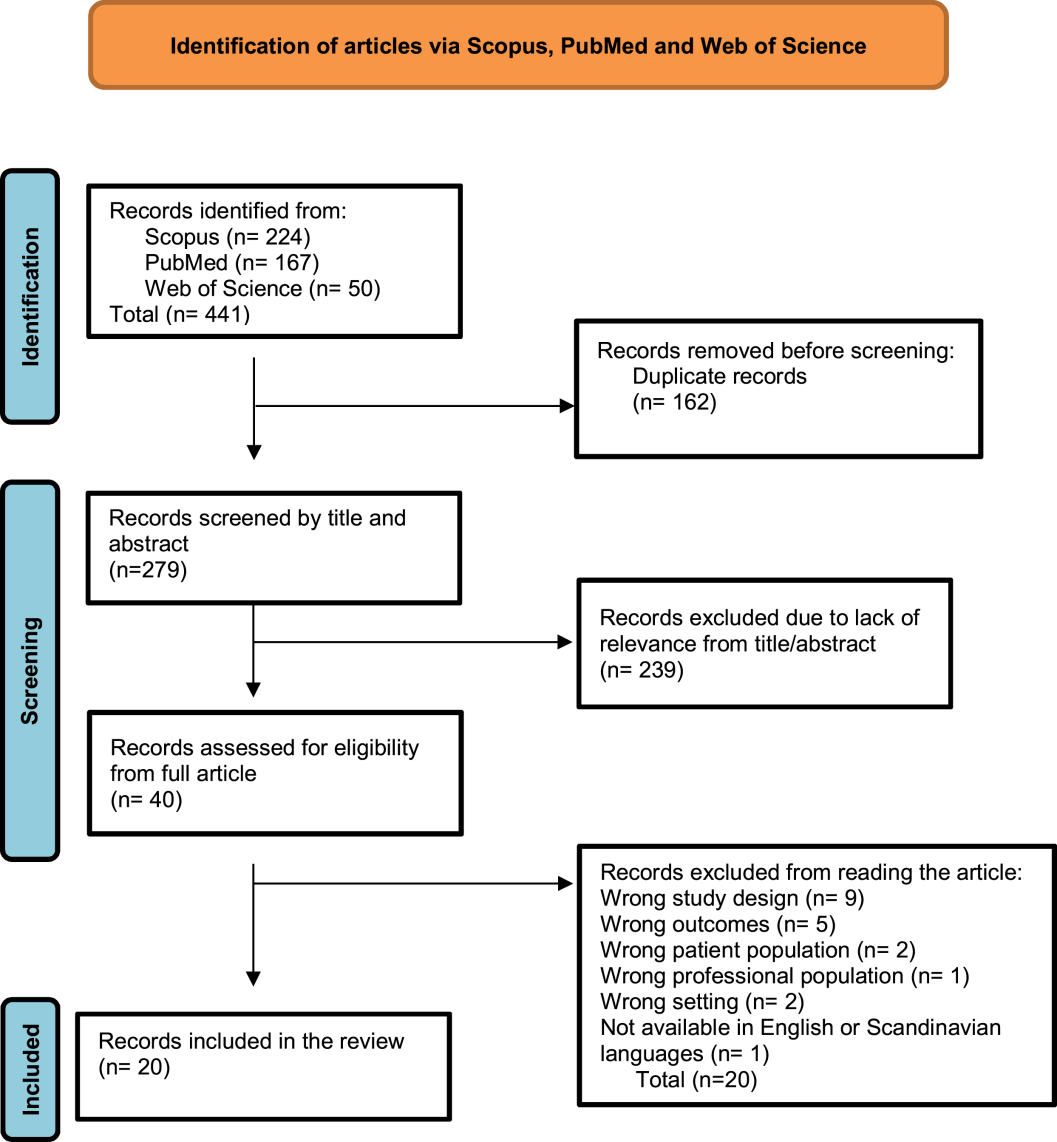
Preferred Reporting Items for Systematic Review and Meta-Analysis (PRISMA 2020) flow diagram for new systematic reviews, which included searches of databases^20^

Twenty articles were included, see Figure 1: PRISMA flow chart. Reference lists of all articles were cross-checked to ensure no relevant literature before 2013 was missed. Articles were assessed for quality using Mixed Methods Appraisal Tool (MMAT). See Supplementary Table S1.

### Data synthesis

Both quantitative and qualitative study designs covering identification of ARVI in general practice were included. Quantitative data are described alongside qualitative findings under common themes. The authors followed a process of thematic qualitative synthesis that moves from initial coding and then more analytical themes.^21^ The process was iterative, and codes and themes were refined and developed throughout the analysis process (See supplementary Table S2 for author contributions).

## Results

In total, 279 articles were screened for title and abstract. Forty articles were full-text screened. Twenty articles were included in the analysis^22–41^ (see Supplementary Table S3).

The articles were qualitative (*n* = 4),^23–25,39^ mixed methods (*n* = 4),^22,26,34,38^ and quantitative (*n* = 12).^27–33,35–37,40,41^ Studies were from Australia (*n* = 7),^22,25,30–32,35,36^ UK (*n* = 6),^23,24,26,33,37,38^ Canada (*n* = 2),^28,34^ New Zealand (*n* = 2),^39,41^ the Republic of Ireland (*n* = 1),^29^ Germany (*n* = 1),^40^ and Norway (*n* = 1).^27^

### General practice screenings and referrals

In the included articles, GPs do not diagnose ARVI or eye diseases, but refer onwards based on suspicion of disease.^22,23,27–34,37,38^ Referrals from GPs appear to be forwarded, often without assessing specific symptoms or risk factors. This can be exemplified by Basilious *et al* who found that fewer GPs than expected by ophthalmologists were aware of risk factors for glaucoma such as African descent (46%), chronic corticosteroid use (84%), and more than 5-year intervals between eye examinations.^28^

Three articles reported on the quality of referral notes from GPs and optometrists to ophthalmologists and found room for improvement.^31–33^ For example, Fung *et al* found that 71% of GP referrals included details about the patient’s medication, compared with only 57% of optometrist referrals.^33^

Most articles focused on DR.^22–25,27,29,30,32,34,35,40^ Diabetes mellitus (DM) is typically managed in general practice, which matches our finding of DR to be more integrated in general practice compared with other eye diseases, with articles describing general practice staff making routine referral to DR screening.^27,29^ Grimshaw *et al* found that GPs had high baseline levels of intention to advise patients to attend DR screening.^34^ Bakke *et al* reported how practices which used a structured diabetes form, and involved ancillary staff in screening procedures for diabetic microvascular complications, recorded more eye examinations.^27^ A high workload in general practice and difficulties with reimbursement of new initiatives were identified as barriers.^27,40^

### Collaboration between general practices and other health professions

Collaboration was a recurrent theme in the included articles. Internally in a general practice setting, the general practice nurses were highlighted as a relevant workforce to perform DR screenings.^24–27,29,37^ Crossland and Jackson discussed how general practice nurses could take on the role of driving DR screening^22^ and Lindenmeyer *et al* used an example of general practice nurses who had integrated vision screening as part of the preparatory work along with measuring blood pressure.^24^ General practice nurses can be part of securing the continuity of care in eye health,^26,27^ especially in rural areas.^35^ Hipwell *et al* applies a more general terminology of practice staff and includes healthcare assistants as important in DR screenings,^23^ but the wider general practice personnel is not further mentioned in the included articles.

Three articles argue that general practice is not equipped to perform eye examinations,^25,28,41^ making collaboration between general practice and other health professions or sectors important.^23,24,26–37,40^ Five articles reported collaboration with optometrists,^22,23,26,30,37^ 10 articles with ophthalmologists in both primary and secondary sector,^22,27,28,30–33,35,37,40^ and five with other professions such as health workers and screeners.^22–24,29,35^ The collaboration is mostly found through referrals.^22,23,27–34,37^

A close relationship to the local optometrist and a partnership with an ophthalmologist is exemplified as relevant in screening and detection of DR.^22,30^ Not creating competition between professions to avoid rigid treatment was further emphasised.^22^ Holdsworth *et al* found collaboration was present in using general practice as a setting for an optometrist-led glaucoma service check.^26^

### Potential in general practice for detecting ARVI

Several articles argued that much can be done to optimise referrals, collaboration, and role of general practice in detecting ARVI. This was especially prominent in articles focusing on DR.

Five articles showed perspectives on eye examinations performed in general practice.^22,25,30,35,40^ In Watson *et al*, GPs expressed how they found ophthalmologists and optometrists to be better suited to perform DR screenings owing to barriers in general practice setting such as costs of retinal cameras, time constraints, and lack of skills to diagnose DR.^25^ The GPs suggested enabling strategies, such as increased access to continuing professional development, subsidising the costs of retinal cameras, and involving specialists in retinal photography, to promote a wider implementation of DR screening in primary care settings.^25^ Wewetzer *et al* argued for the potentials in implementing AI-assisted DR screening devices in general practice to broaden the scope of care in general practice and appear as a modern medical practice, and engage various disciplines among the practice staff.^40^

Crossland *et al* found the use of retinal photography by GPs effective in detecting DR, and inclusion of DR screening in general practice into the Annual Cycle of Care to be highly effective.^30^ One article argued that existing DR screening could be improved, and that GPs hold an important role in screening uptake.^27^ Here, the potentials in more structured and routine-based DR screening were reported.^23,24,30^

Glasson *et al* demonstrated that involving local health workers in remote areas, who sent photos to GPs for grading and referrals, helped increase screening rates from 16.3% to 66.3%.^35^ Lindenmeyer *et al* found that general practice encouraged patients to attend screening as routine care.^24^

Three articles indicated that general practice could play a more active role in identification of eye diseases other than DR.^26,36,41^ In one qualitative study, participants said they would first see their GP because they felt comfortable and familiar with their service.^39^ Holdsworth *et al* found potential in general practice as a setting for a glaucoma check service.^26^ Singh *et al* found that GPs could use smartphone-based ophthalmoscopes to meet the challenges in direct ophthalmoscopy.^41^ The difficulty in using direct ophthalmoscopy was reported by Basilious *et al* as only 28% of GPs were comfortable with performing the procedure.^28^

## Discussion

### Summary

Only 20 articles were identified in covering the role of general practice in the identification of ARVI.

The included studies mostly focused on DR screening. Only a few articles focused on the identification of other eye diseases such as glaucoma and AMD, or the issue of ARVI without a specific diagnostic focus. In this review, we have searched for research conducted in welfare states with a strong primary care sector. Denmark, with a population of 5.9 million (including 1.6 million people aged >60 years), serves here as an example. According to the Danish Medical Council, approximately 450 new cases of diabetic macular edema requiring treatment are diagnosed each year, while 15 000 individuals with wet AMD received anti-vascular endothelial growth factor (anti-VEGF) injections in 2022.^42^ Additionally, the prevalence of glaucoma among individuals aged >50 years was reported to be 3.76% in 2011.^43^

The literature’s primary focus on DR is likely owing to DM being a chronic disease, which is routinely followed in general practice, while AMD, glaucoma, and cataract are eye diseases primarily examined and treated solely by ophthalmologists.

The potential of general practice to be more involved in detection of chronic eye diseases was identified. For example, through better knowledge on risk factors for glaucoma,^28,31^ by following routine screening procedures for DR,^27,35^ and by using alternatives to direct ophthalmoscopy,^35,41^ including AI-assisted devices,^40^ which can be especially relevant in rural areas with limited access to ophthalmology services.^35^

### Strengths and limitations

To our knowledge, this review is the first to assemble the literature on identification of ARVI including chronic eye-diseases in general practice. The PRISMA guideline was followed ensuring a systematic approach. Three databases with both qualitative and quantitative literature were searched. Careful considerations and preparation of the comprehensive search string were made to find existing relevant literature.

Articles from different countries with different healthcare systems were included. This made comparison difficult, but provided an insight on identification of ARVI in general practice in countries with a strong primary care sector. The search was limited to an 11-year period to focus on current tendencies in the research field. However, a potential consequence of this is that relevant articles before 2013 were missed. To minimise the risk of missing key articles published before 2013 all reference lists in the included articles were cross-read. Our search string focused on ARVI, including chronic eye diseases, and did not include acute eye diseases in the search string. This matches our aim to highlight knowledge on ARVI, which might not present clear symptoms and how these are managed in general practice. A limitation is that articles on acute-eye issues were missed owing to being out of scope.

### Comparison with existing literature

In cross-reading the reference lists of the included articles, 11 articles from before 2013 were cited in a minimum of three of the 20 included articles.^44–54^ Five were from Australia^44,45,48,51,52^ and two from the UK.^46,49^ Ten articles focused on DR prevalence, identification, and screening programmes.^44–53^ Five specifically on DR screening in a general practice setting.^44,45,51–53^ One presented the global data on vision impairment in 2002.^54^ The findings correspond with the 20 included articles.

The predominance of articles from the UK and Australia resembles the geographical representation in a Cochrane review from 2018.^55^ The review assessed the effects of 10 randomised controlled trials (RCT) investigating community vision screening of people aged ≥65 years and not belonging to a particular risk group. The authors did not find statistically significant evidence in favour of community vision screening. Similar unclear findings regarding the evidence on eye screening programmes were found in the US Preventive Services Taskforce on screening for impaired visual acuity in older adults.^56^ Varadaraj *et al* were critical of the taskforce findings, highlighting the challenges in the US related to a weak primary health sector and the eye health inequalities associated with this situation.^57^ The US was not included as a geographical area in our search owing to the weak general practice function.

Of the included articles only Sim *et al* ^37^ focused on AMD. Given the substantial patient burden of AMD and financial societal impact,^1^ we had expected a higher number of articles addressing detection of AMD. Yip *et al* found AMD to be associated with economic inability owing to visual disability and called for a greater public health focus on eye health.^58^ Piano *et al* underlined, how general practice can be key in helping people living with dementia in accessing eye care, emphasising the importance of vision when managing dementia.^59^

### Implications for research and practice

The limited number of studies identified emphasises that detection of ARVI is currently an under-researched area in a general practice setting.

ARVI can have significant negative consequences on older patients’ overall health, including higher prevalence of depression,^10^ making it a relevant sensory impairment to address. Overall, the included articles suggest that general practice holds a potential role in identification.^26–28,35,40,41^ Identification of DR could be improved by implementing routines for regular checks and by using a structured electronic diabetes form or similar tool in general practice.^23,24,27,30,40^ In the literature, general practice nurses are highlighted as drivers for implementing such vision screenings.^24–27,29,37^ Detection of ARVI could thus be assigned to the wider practice team in general practice. The articles included did not provide detailed information regarding this aspect, but it is an interesting subject to investigate further; that is, through investigating the effects on detection of DR through distribution of tasks in general practice.

This review suggests further research on identification of glaucoma and AMD in general practice. These chronic eye diseases have high prevalence, are generally asymptomatic, and both conditions are associated with a significant patient burden.^58,60^ If general practice staff are properly prepared to recognise indications of the conditions, effective treatment options exist and early detection can contribute to saving patients' vision. More evidence is therefore needed to guide future policy changes and educational reforms.

## Data

The dataset relied on in this article is available from the corresponding author on reasonable request.
